# Synergistic Effect in Neurological Recovery via Anti-Apoptotic Akt Signaling in Umbilical Cord Blood and Erythropoietin Combination Therapy for Neonatal Hypoxic-Ischemic Brain Injury

**DOI:** 10.3390/ijms222111995

**Published:** 2021-11-05

**Authors:** Jee In Choi, Joo-Wan Choi, Kyu-Ho Shim, Jin Seung Choung, Hyun-Jin Kim, Hye Ryeong Sim, Mi Ri Suh, Joo Eun Jung, MinYoung Kim

**Affiliations:** 1 Department of Rehabilitation Medicine, CHA Bundang Medical Center, CHA University School of Medicine, Seongnam 13496, Korea; cji-012@daum.net (J.I.C.); suhmiri@gmail.com (M.R.S.); 2 Rehabilitation and Regeneration Research Center, CHA University School of Medicine, Seongnam 13488, Korea; bubukkum@naver.com (J.-W.C.); kyuhoshim@gmail.com (K.-H.S.); choungjs@gmail.com (J.S.C.); khjj4188@naver.com (H.-J.K.); ryeong1436@naver.com (H.R.S.); 3 Department of Neurology, University of Texas Health Science Center at Houston, McGovern Medical School, Houston, TX 77030, USA; garvy76@gmail.com

**Keywords:** cord blood cell therapy, erythropoietin, hypoxic–ischemic brain injury, neurobehavioral recovery, anti-apoptosis, Akt signaling pathway

## Abstract

Our previous clinical studies demonstrated the synergistic therapeutic effect induced by co-administering recombinant human erythropoietin (rhEPO) in human umbilical cord blood (hUCB) therapy for children with cerebral palsy. However, the cellular mechanism beyond the beneficial effects in this combination therapy still needs to be elucidated. A hypoxic–ischemic encephalopathy (HIE) model of neonates, representing cerebral palsy, was prepared and randomly divided into five groups (hUCB+rhEPO combination, hUCB, and rhEPO treatments over HIE, HIE control, and sham). Seven days after, hUCB was administered intraperitoneally and the rhEPO injections were started. Neurobehavioral tests showed the best outcome in the combination therapy group, while the hUCB and rhEPO alone treatments also showed better outcomes compared with the control (*p* < 0.05). Inflammatory cytokines were downregulated by the treatments and attenuated most by the combination therapy (*p* < 0.05). The hUCB+rhEPO treatment also showed remarkable increase in phosphorylation of Akt and potentiation of anti-apoptotic responses with decreased Bax and increased Bcl-2 (*p* < 0.05). Pre-treatment of MK-2206, an Akt inhibitor, for the combination therapy depressed the anti-apoptotic effects. In conclusion, these findings suggest that the therapeutic effect of hUCB therapy might be potentiated by co-administration of rhEPO via augmentation of anti-inflammatory and anti-apoptotic responses related to the phosphorylation of Akt.

## 1. Introduction

Cerebral palsy (CP) is a disabling disorder developed during the early childhood period which means difficulty of recovery from insult in the immature brain [1]. The major cause of the brain lesion is known to be hypoxic–ischemic encephalopathy (HIE) [2]. It is distinguished from similar damage in the mature brain by exhibiting more exacerbated inflammation and apoptosis as enlightened in previous research [3]. While its main clinical representation is motor deficits, the patients frequently have other neuronal dysfunctions involving sensation, perception, cognition, and communication throughout their lives [4,5]. Despite the advances in perinatal care, there are no effective treatment strategies to circumvent perinatal HIE in humans [6]. Lately, stem cell therapy has been recognized as a promising strategy that functionally enhances recovery from brain injury [7,8,9].

Human umbilical cord blood (hUCB) has been used clinically for almost 30 years for hematopoietic purposes and is also used to treat metabolic disorders involving cerebral dysfunction [10]. The mononuclear cell fraction of hUCB that contains numerous cell types, including lymphocytes and several stem cells, is expected as being involved in the therapeutic effects [11]. Previous studies testing hUCB cell therapy have demonstrated excellent potential to protect or repair brain injury [12,13,14]. As therapeutic mechanisms, anti-apoptotic and anti-inflammatory actions of the hUCB mononuclear cells that accompany neurogenesis and angiogenesis have been reported [10]. Recent clinical research has suggested that these neurodevelopmental disorders may be ameliorated by hUCB in children with CP [12,13,15]. While other sources of therapeutic cells are also on clinical trial, from an efficacy perspective, cell therapies have failed to reach clinical expectations [7,16].

Up until now, measures to potentiate the efficacy of cell therapy have been attempted, such as genetic manipulation to increase brain-derived neurotrophic factor, which has shown potential therapeutic effects [17,18]. Since most of these attempts require further research before being applied in the clinical setting to ensure safety, the present study team found recombinant human erythropoietin (rhEPO) as a potentiator for hUCB-administrating treatment. rhEPO is actively used in clinics to induce erythropoiesis, and due to its neuroprotective characteristics, it has been under investigation for a long time to overcome neurological diseases [19,20]. rhEPO-induced neuroprotection involves several downstream signaling pathways of the erythropoietin (EPO) receptor. For instance, homodimerization on rhEPO binding allows for the autophosphorylation of rhEPO receptor-associated Janus kinase (JAK)2. The phosphorylation of JAK2 leads to the activation of several signaling pathways, including signal transducer and activator of transcription (STAT) 5, the extracellular-signal-regulated kinase (ERK)/mitogen-activated protein kinase (MAPK), and phosphoinositide 3-kinase (PI3K)/Akt [21]. The roles of these signaling pathways induced by rhEPO exert neuroprotective and anti-apoptotic effects [22]. Therefore, rhEPO may be a promising therapeutic agent to reinforce hUCB treatment for HIE.

Our previous clinical results showed a superior outcome in motor function restoration when the rhEPO was co-administered in hUCB therapy for patients with CP [12,15]. An in vivo stroke model study also revealed the potentiation of hUCB therapy by co-administration of rhEPO with findings of enhanced neurogenesis and angiogenesis, resulting in better neurobehavioral recovery than the administration of hUCB only [23]. To understand the mechanism of the synergistic effect of hUCB and rhEPO in HIE, the authors hypothesized the existence of a signaling pathway that is shared by the treatments. In brain injury, activation of PI3K/Akt signaling is known to be a classical signal transduction pathway that resists against neuronal death after HIE by inactivating the apoptotic response [24]. Experiments using a rat model of CP showed the involvement of the PI3K/Akt signaling pathway with the anti-apoptotic effect as a therapeutic mechanism [25]. Furthermore, it was reported that hUCB was attributed to secreted soluble factors, many of which are known activators of the Akt signaling transduction pathway [26]. Taken together, Akt signaling may exert therapeutic effects as a common route of action.

To our knowledge, the corresponding mechanism of the synergistic effect of hUCB and rhEPO has not been investigated in a controlled condition. In this study, the authors proposed to find the significant pathway that plays an important role in the synergistic effect of hUCB and rhEPO when they were applied for HIE (1 h at 8% O_2_ and 92% N_2_) of an immature mouse (P7). Assays for apoptosis and neuroregeneration were also conducted along with neurobehavioral outcome tests. Discovering the key molecular pathway in neuronal recovery in HIE could be significant for the further development of efficacious therapy for CP.

## 2. Results

### 2.1. Neurobehavioral Outcome by rhEPO or hUCB or hUCB+rhEPO Combination Treatment in HIE

To compare the effects of each treatment, five groups were randomly generated: combination of hUCB and rhEPO (UCB+EPO group), hUCB alone (UCB group), rhEPO alone (EPO group) treatments over HIE, the HIE without treatment control group, and the normal sham control group. The mice were isolated from their mother mouse at postnatal 3 weeks (P21) and were reared by gender. To assess neurobehavioral outcomes by rhEPO, hUCB, and combination of hUCB and rhEPO treatments, we performed the modified neurological severity score (mNSS) [27,28] and cylinder tests [27,29] at 4 weeks after the hUCB administration (P42).

**Figure 1:**
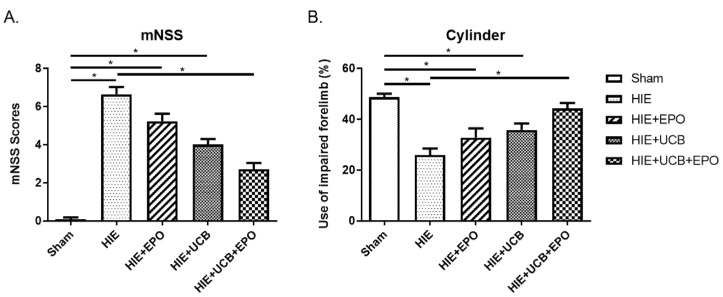
Neurobehavioral improvement after rhEPO, hUCB, and combination treatment of rhEPO and hUCB in HIE model. (**A**) The graph represents mNSSs (0 (normal)~14 (severe injury)) in each group. (**B**) The graph represents the percentage of impaired forelimb use in each group. Sham (normal); HIE (hypoxic–ischemic encephalopathy); HIE+EPO (500 IU/kg of rhEPO, intraperitoneal injection for five consecutive days from 7 d after HIE); HIE+UCB (3 × 10^7^/kg of hUCB, intraperitoneal injection once at 7 d after HIE); HIE+UCB+EPO (combination of hUCB and rhEPO treatment on HIE). Data shown as the mean ± standard error of mean. *n* = 9 per group. * *p* < 0.05 ((**A**) Kruskal–Wallis; (**B**) one-way ANOVA).

The neurobehavioral outcome revealed neurological impairment by HIE by both the mNSS and cylinder test (*p* < 0.05). While the EPO group and UCB group showed impaired status compared to the sham control group (*p* < 0.05) without difference from HIE, only the UCB+EPO group showed recovery from the neurological deficits by both the mNSS and cylinder test. The mNSS was lower in the UCB+EPO group compared to the HIE group, which indicates ameliorated neurological dysfunction (*p* < 0.05) (Figure 1A). In addition, the cylinder test score of the UCB+EPO group was higher than the HIE group, which indicates improvement in the paralyzed forelimb (*p* < 0.05) (Figure 1B).

### 2.2. Neuroprotective Effect by rhEPO or hUCB or hUCB+rhEPO Combination Treatment in HIE

**Figure 2:**
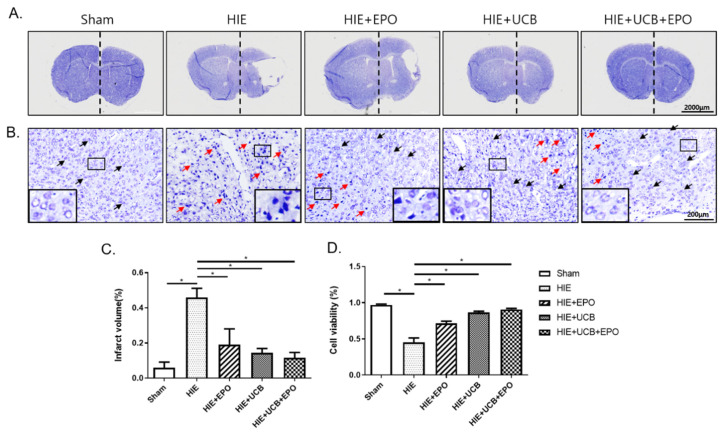
Attenuation of brain damage and prevention of neuronal cell death in brains of HIE model in each treatment group. (**A**) Whole brain sections were stained with cresyl-violet and (**C**) the graph depicts the total volume of the infarct size in the affected hemisphere, which was estimated in comparison with that in the contralateral side by the ImageJ program. (**B**) The enlarged images show the morphology of the neuronal cell. Normal cells are indicated with black arrows and abnormal cells with red arrows. (**D**) The graph depicts the cell viability by the percentage of neuronal cells after each treatment group. Data shown as the mean ± standard error of mean. *n* = 3 per group. * *p* < 0.05 (one-way ANOVA).

Next, we tested whether hUCB and rhEPO can protect against neuronal cell death from HIE. At 4 weeks after hUCB administration (P42), brain tissues were isolated and stained using cresyl-violet solution to assess cerebral volume loss by infarction at the lesion side. There were no detectable brain infarct lesions in the sham group. In comparison with the contralateral brain, after measuring each side’s brain volume, lost cerebral volume was calculated. Loss of cerebral volume in the ipsilesional side reached about 50% in the HIE group, which was the most profound compared with all of the other groups (*p* < 0.05). The cerebral infarction volume was gradually reduced in order by rhEPO alone, hUCB alone, and combination administration without significant differences among the therapy groups (Figure 2A,C).

To assess neuronal cell death in the brain tissue, caused by HIE, histological analysis was performed. In the magnified images, the neuronal cells of the sham control group exhibited normal morphologic properties with round nuclei. Whereas cells were shrunken and nuclei were irregularly condensed in the HIE group, all treatment groups showed recovery-pattern cells with normal morphologies; however, some condensed nuclei coexisted in them (Figure 2B). The number of neuronal cells at the cortex near the site of ischemic lesion was counted with the samples for determination of cell viability. It revealed better survival of neuronal cells in the treated groups than in the HIE group (*p* < 0.05) with a marked neuroprotective effect in the UCB+EPO group (*p* < 0.05) (Figure 2B,D).

### 2.3. Reduction of Pro-Inflammatory Gene Expression by rhEPO or hUCB or hUCB+rhEPO Combination Treatment in HIE

**Figure 3:**
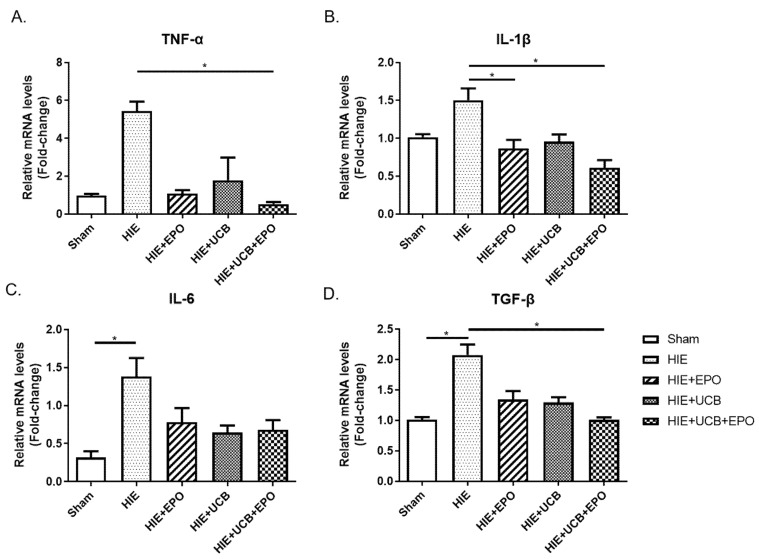
Pro-inflammatory cytokine gene expression was decreased in each treatment group. (**A**–**D**) The mRNA expression of pro-inflammatory cytokines (TNF-α, IL-1β, and IL-6) and TGF-β was detected by qRT-PCR. Data shown as the mean ± standard error of mean. *n* = 4 to 6 per group, * *p* < 0.05 (Kruskal–Wallis).

The regulation of cytokines involved in the inflammatory response has emerged as an important therapeutic indicator in HIE. We assessed the gene expressions of inflammation-regulating cytokines in the brain tissue 7 days after the administration of hUCB (P21) using quantitative real time PCR (qRT-PCR). The expression level of tumor necrosis factor-α (TNF-α), a representative pro-inflammatory marker, was significantly reduced in the UCB+EPO group (*p* < 0.05) (Figure 3A). Similar results were found in the gene expressions of interleukin-1β (IL-1β) with lower level in the UCB+EPO group than the HIE group (*p* < 0.05) (Figure 3B). The level of IL-6 increased in the HIE group (*p* < 0.05), while it showed a tendency to decrease with all treated groups without significance (Figure 3C). The gene expression of transforming growth factor-β (TGF-β) which is known to induce apoptosis through TGFβ1/Smad signaling in HIE, appeared to be elevated by HIE (*p* < 0.05) and significantly downregulated in the HIE+EPO group compared with the HIE group (*p* < 0.05) (Figure 3D). However, the levels of IL-2 and IL-10 did not show differences among the groups (data not shown). The level of tyrosine kinase B (TrkB), which is a receptor for brain-derived neurotrophic factor (BDNF) and the Nkx2.1 known to be involved in brain development, showed a tendency of increment by hUCB or rhEPO without statistical significance (data not shown).

### 2.4. Up-Regulation of Anti-Apoptotic Effect by hUCB+rhEPO Combination Treatment in HIE

**Figure 4:**
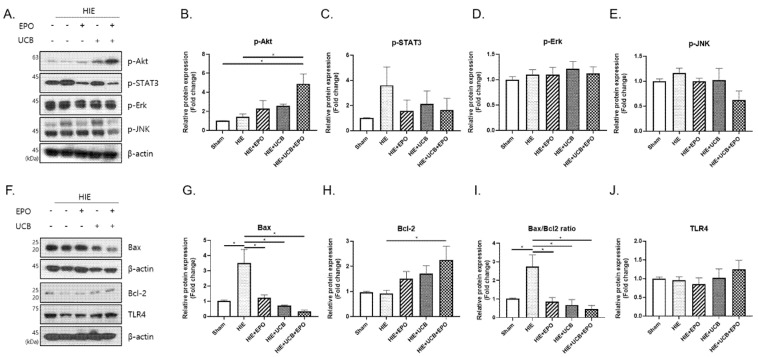
Each treatment group up-regulated the anti-apoptotic effect via Akt signaling. (**A**) The levels of *p*-Akt, *p*-STAT3 (S727), *p*-Erk and *p*-JNK were detected using Western blotting to observe the consecutive changes after administration. (**B**–**E**) The graphs depict the band intensity by the ImageJ program for the band of image A. (**F**) The levels of related apoptosis proteins (Bax and Bcl-2) and TLR4 after hUCB and rhEPO treatment. (**G**,**H**,**J**) The graphs depict the band intensity and (**I**) represents the Bax/Bcl-2 ratio. Data shown as the mean ± standard error of mean. *n* = 3 to 8 per group, * *p* < 0.05 ((**B**,**C**,**J**) Kruskal–Wallis; (**D**,**E**,**G**–**I**) one-way ANOVA).

In order to investigate the molecular mechanism of the synergistic effect by hUCB and rhEPO combination therapy, several proteins such as Akt, STAT3, Erk, JNK, and toll-like receptor 4 (TLR4), which might be involved in hUCB- or rhEPO-mediated signaling pathways, were examined by Western blot in the brain tissue of the HIE model at P21. The other proteins did not show significant findings and only Akt, a serine/threonine-specific protein kinase, showed a remarkable response (Figure 4). It is a key signal transduction pathway that has an important role in the regulation of apoptosis and cell survival. The results showed higher level of Akt phosporylation in the UCB+EPO group compared to the HIE group and sham group (*p* < 0.05). (Figure 4A,B). Further experiments to assess the effects on apoptosis revealed a possible anti-apoptotic role of Akt by preventing the release of Bax, which was increased by HIE. The expression level of Bax was lower in the UCB+EPO group compared with all the other HIE afflicted groups (*p* < 0.05) (Figure 4F,G). As a concordant finding, the protein expression of Bcl-2, which inhibits apoptosis and promotes cellular survival, was significantly upregulated only in the UCB+EPO group (*p* < 0.05) (Figure 4F,H). Accordingly, the Bax/Bcl-2 ratio which was increased by HIE showed lowered values in all treatment groups (*p* < 0.05) (Figure 4F,I). In addition, the phosphorylation levels of STAT3, which showed a tendency of increment in the HIE group, showed a tendency to decrease in the treatment groups, seeming to represent an activated downstream signal of IL-6 (Figure 4A,C).

### 2.5. The MK-2206 Inhibits the Anti-Apoptotic Effect by Akt Phosphorylation in the hUCB+rhEPO Combination Treatment Group

**Figure 5:**
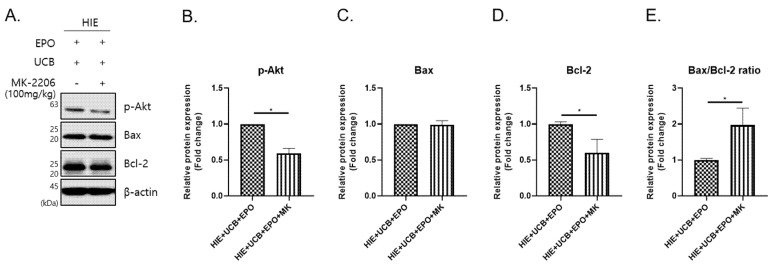
Anti-apoptotic effect inhibits by MK-2206 (Akt inhibitor) in hUCB and rhEPO combination treatment. (**A**) The levels of *p*-Akt, Bax, and anti-apoptotic protein, Bcl-2, by phosphorylation of Akt were detected using Western blotting. (**B**–**D**) The graphs depict the band intensity by imageJ program for band of image A, and (**E**) represent Bax/Bcl-2 ratio. Data shown as the mean ± standard error of mean. *n* = 4 to 5 per group, * *p* < 0.05 (one-way ANOVA).

Given the fact that the Akt pathway is activated in the hUCB and rhEPO group, we examined if the Akt-mediated anti-apoptotic pathway is inhibited in the HIE brain pretreated with Akt inhibitor, MK-2206 2HCl (MK-2206) in hUCB and rhEPO combination therapy. MK-2206 was injected before the administration of hUCB and rhEPO (Figure 8). As a result, Akt phosphorylation was robustly reversed by MK-2206 pre-treatment in the brain of HIE mice that received hUCB and rhEPO combination therapy (*p* < 0.05) (Figure 5A,B). And the level of Bcl-2, which was significantly increased by hUCB and rhEPO combination therapy, was also robustly reversed by MK-2206 pre-treatment (*p* < 0.05) (Figure 5A,D). Importantly, the Bax/Bcl-2 ratio was reversed by MK-2206 treatment in the HIE brains of mice that received combination therapy (*p* < 0.05) (Figure 5A,E). This finding indicates that hUCB and rhEPO combination therapy upregulates the anti-apoptotic factor, Bcl-2 by Akt phosphorylation, which may protect the brain against HIE injury and promote cell survival.

### 2.6. Enhancement of Neuroregenerative Capability by rhEPO or hUCB or hUCB+rhEPO Combination Treatment in HIE

**Figure 6:**
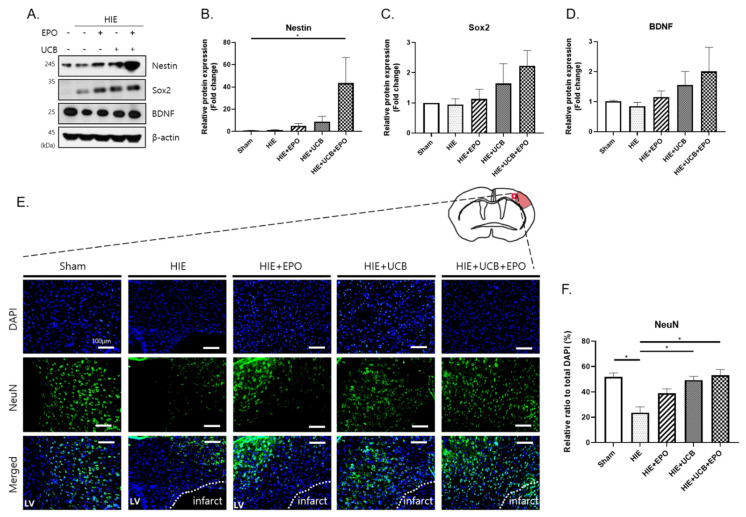
Neuroregenerative capability by the combination treatment hUCB and rhEPO group was increased in the HIE model through a synergistic effect. (**A**) Up-regulation of the levels of Nestin, Sox2, and pro-BDNF (32 kDa) protein was shown by Western blot. (**B**–**D**) The graphs depict band intensity. (**E**) Neuroprotection was observed through immunohistochemistry staining for NeuN in the peri-infarct cortex of the HIE brain. White bar indicates 100 µm. (**F**) The graph depicts the quantification of the number of NeuN (+cells. Data shown as the mean ± standard error of mean. *n* = 3 per group. * *p* < 0.05 ((**B**,**D**) Kruskal–Wallis; (**C**,**F**) one-way ANOVA).

A Western blotting assay was performed to demonstrate early neuroregenerative responses by rhEPO, hUCB, or their combination in HIE, using the brain tissue extracted 7 d after each treatment. The level of Nestin was increased in the UCB+EPO group compared with the sham group (*p* < 0.05) (Figure 6A,B). In addition, the level of Sox2 showed a gradually tendency to increase in each treatment group compared with the HIE group, the most increase being in the UCB+EPO group without significance (Figure 6A,C). These results indicate that the combination treatment possibly boosts the neurogenerative effect of hUCB therapy. The level of pro-BDNF also showed a tendency of increment in the UCB+EPO group (Figure 6A,D). In order to confirm the neurorestoring effect by combination treatment in the long-term aspect, immunohistochemistry analysis with NeuN, a mature neuron marker, was performed for a sectioned brain sample extracted 28 d after the treatment. While NeuN (+) cells were hardly observed in the HIE cortex, the UCB group and UCB+EPO group showed significant increase of NeuN (+) cells at the peri-infarct area in the cortex (*p* < 0.05) (Figure 6E,F).

## 3. Discussion

After an injury to the central nervous system, rapid cellular responses follow during the acute stage in the neural tissues including apoptosis and inflammation which may impose further neural damage [30]. In the developing brain, the impact of a brain injury remains persistent for more than several years, which is different from the response in a mature brain [31]. Therefore, children with CP caused by an acquired brain injury that occurred during the perinatal period have difficulty in acquiring higher motor function even after many years and might even experience further functional decline [32]. In order to overcome these obstacles, various studies using stem cells have been reported. However, due to limited therapeutic effects of the cell therapy, efforts to potentiate the efficacy have been exerted [8].

Our previous clinical studies revealed the efficacy of the synergistic effect of combination therapy, as hUCB with rhEPO, for these children might be useful as therapeutic measures. [12,15]. In the present study, we aimed to confirm the synergistic effect of hUCB and rhEPO treatments and to determine the mechanism underlying the synergy. A neonatal HIE model after the acute stage was used to mimic the childhood period of CP. This experiment also showed the augmented therapeutic efficacy of hUCB by co-administrating rhEPO in neurobehavioral assessments, while the hUCB- or rhEPO-alone treatments also brought better outcomes than the non-treated groups. hUCB, a source of hematopoietic progenitor, has been used for bone marrow transplantation and has also been under investigation for the purpose of neurorestoration due to its ability to attenuate inflammation and its neuroprotective capacity [33]. Clinical applications of hUCB treatment have been attempted for neuronal diseases including stroke and CP [33,34]. According to a previous clinical trial for children with CP, the administration of hUCB resulted in better outcomes of motor development [13]. However, in another longer follow-up, study results showed insufficient efficacy by hUCB treatment alone. By co-administering rhEPO, the therapeutic efficacy became more potent without a harm issue [15]. rhEPO is known to play a role in protecting nerve cells in spinal cord trauma via counteracting secondary injury [35]. Neonatal animal studies have shown that immediate treatment of rhEPO after HIE can be neuroprotective and restorative [36]. In addition, in a clinical trial on children with CP, the administration of rhEPO brought improvements in motor function compared with the control group [37].

The combination treatment of hUCB cells and rhEPO brought the best neurobehavioral outcomes in our previous stroke animal study when compared with a single treatment with hUCB or rhEPO, which also showed some efficacies. The treatments induced neurogenesis and angiogenesis with the biggest potency in hUCB and rhEPO co-administration [23]. Likewise, in the present research, scores of neurobehavioral tests showed best outcomes in the UCB+EPO group compared with other HIE-afflicted groups (Figure 1). The mNSS and cylinder test revealed significant therapeutic efficacy only in the UCB+EPO group and the other therapy group failed to show enough potency. Other outcomes including the cerebral infarct volume and the cell viability revealed some therapeutic effects of hUCB and rhEPO with relatively best results by combination therapy. Therefore, we can confirm the potentiating therapeutic effect of rhEPO co-administration in hUCB treatment in neonatal HIE.

As therapeutic action mechanisms involved in hUCB and rhEPO treatment, anti-inflammatory and anti-apoptotic responses were considered and investigated. Previous research reported that the transplantation of hUCB or hUCB-derived mesenchymal stem cells (hUCB-MSCs) suppressed inflammation and neuronal apoptosis in brain injury [10,38]. In the hUCB-MSC-received group, the protein expressions of IL-1β, IL-6, and TNF- α were reduced, showing lower apoptotic cells than the non-treatment group [38]. The other study demonstrated a decrement of brain edema and pro-inflammatory cytokine expressions such as IL-1β and TNF-α by rhEPO treatment [39]. In the present study, two weeks after HIE induction, the gene expressions of IL-6 and TGF-β were elevated and those of TNF-α and IL-1β showed a tendency of elevation in the HIE brain tissue. When the hUCB and rhEPO combination treatment was given one week after HIE induction, the gene expressions of TNF-α, IL-1β, and TGF-β were downregulated on P21, while each single treatment brought similar trends with weak intensity. The treatments reduced IL-6 gene expression without significance. Collectively, the therapeutic effect of rhEPO co-administration in hUCB treatment potentiated an anti-inflammatory effect, possibly associated with the reduced activities of pro-inflammatory cytokines.

As for the anti-apoptotic response, the Akt signaling pathway was found to be involved as a key mechanism, especially in the synergistic effect of hUCB and rhEPO combination treatment. In the present study, hUCB and rhEPO co-administration markedly increased phosphorylated Akt, while hUCB or rhEPO treatment alone also showed an elevated level with lower potency without significance. The PI3k/Akt signaling pathway plays a role in promoting neuronal survival in HIE by suppressing the inflammation response [40]. Previous reports revealed the induction of PI3k/Akt signal transduction either by hUCB or rhEPO administration [21,26]. The signaling pathway regulates cell growth and survival through various downstream pathways including Bax or Bcl-2-related apoptosis [41], which might have significance in brain injury. We also demonstrated that the combination hUCB and rhEPO therapy modulated Bax and Bcl-2 among several downstream-inducing anti-apoptotic effects. The level of Bcl-2 was elevated only in the UCB+EPO group and the Bax/Bcl-2 ratio was depressed in all treatment groups. When MK-2206, an Akt inhibitor, was simultaneously administered to the hUCB and rhEPO co-administered group, the anti-apoptotic effect through *p*-Akt was diminished. These data suggested that co-administration by hUCB and rhEPO is responsible for the synergistic anti-apoptotic effect through the phosphorylation of Akt.

Moreover, the increment of Nestin and Sox2 expressions seemed most remarkable in the UCB+EPO group among the treated groups although Sox2 result did not show significance. NeuN (+) cells were also increased by treating hUCB and rhEPO. Taken together, hUCB and rhEPO combination therapy might exert a neurorestoring effect synergistically. Further investigations would be required to confirm the neuroregenerative potential of the hUCB and rhEPO combination therapy; the observation of increments of neural stem cells in the subventricular zone or hippocampal area in the brain subjected to the therapy; and an experiment of neural stem cell culture under the combination treatment condition to see upregulation of neurogenesis and increase in neuronal differentiation with early neuronal markers.

In this study, the gene expressions of IL-2 and IL-10, which were evaluated to see the effects of Th1 and Th2 in the brain, did not show significant differences among the groups [42]. In addition, the gene expression of TrkB, receptor of BDNF [43], and Nkx2.1 showed trends of increment of these expressions in each administration group without significance.

There are several limitations to consider in this study. First, the observations were made at only two time points (P21, P42) after the HIE insult (P7) and the treatments (P14). It was known that neonatal brain injury progresses rapidly over hours and days after onset and persists into so-called primary, secondary, and tertiary stages of brain injury through several mechanisms, contributing to long-term neurodevelopmental deficits [44]. Therefore, the phase-specific therapeutic mechanisms according to progress after HIE should be elucidated. Second, the PI3K/Akt signal was proposed, as the various transcriptional regulators have a wide range of cellular activities including survival, proliferation, metabolism, and motility of cells [24]. In this study, we focused on the anti-apoptotic effect of Akt, which regulates the expressions of Bax and Bcl-2. Since brain recovery from the injury could be mediated by another aspect of the Akt pathway, such as cell survival and cell proliferation, the combination-therapy-mediated Akt pathway needs to be explored further. In addition, other neuroprotective mechanisms besides PI3K/Akt signaling that are related to neuroprotective properties could have been induced by hUCB and rhEPO [24,45]; although, the phosphorylation of STAT3, Erk, and JNK did not show significant changes in the current study. Finally, the result of the elevated gene expression of *p*-Akt even on P21 in the HIE brain needs to be addressed. Some previous research studies revealed that several models confirmed an increment in *p*-Akt very early, 0.5 to 4 h, after ischemic reperfusion [46,47]. Another investigation found the expression of *p*-Akt re-increased from 24 h after its decrement and sustained a high level until 72 h after ischemic injury [48]. Our study showed a slight elevation of *p*-Akt at 21 d after the HIE injury, and this could have been the result of a re-increased level of *p*-Akt after its decrease. In addition, the treatment of rhEPO or hUCB alone also showed a trend of elevation in *p*-Akt at 21 d compared with the vehicle treatment group; however, the combination treatment of UCB+EPO showed a significant and dramatic elevation in *p*-Akt. Further analysis of the phosphorylation pattern of Akt that changes at each time period is required because the neonatal HIE model still experiences progressing brain development and this condition might affect signaling expressions.

**Figure 7:**
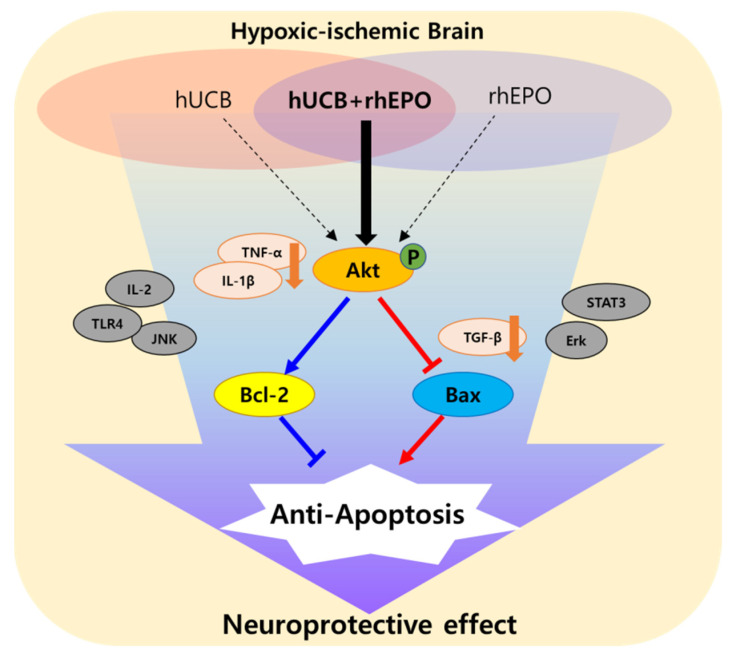
Summary figure. The combination treatment with rhEPO in hUCB therapy improves synergistic neuroprotective effects from the HIE injury through Akt-mediated anti-apoptotic responses in the HIE brain.

In conclusion, our findings in this study demonstrated that the combination treatment with rhEPO in hUCB therapy significantly improves the neurological outcome from the HIE injury through preventing pro-inflammatory reaction and Akt-mediated anti-apoptotic responses in the HIE brain (Figure 7).

## 4. Materials and Methods

### 4.1. Animal

ICR mice (Orient bio, Seongnam, Korea) were acclimated to their environments for 2 days before use. The mice were housed in a temperature-controlled room (22 ± 2 °C), kept at constant humidity (50 ± 10%), and maintained on a 12-h light/dark cycle with ad libitum access to food and water. All experimental procedures involving animals were performed in accordance with the Guide for the Care and Use of Laboratory Animals as adopted and promulgated by the U.S. National Institutes of Health and were approved by the CHA University Institutional Animal Care & Use Committee (IACUC200016).

### 4.2. In Vivo Ischemia Model: Hypoxic–Ischemic Brain Injury (HIE)

At postnatal 7-day, pups, whose sex was randomly selected, were initially anesthetized with 3–5% isoflurane and maintained with 1–2% isoflurane for surgery. Briefly, the adipose tissue of 7-day-old mouse pups was carefully removed using sterilized forceps and the unilateral right carotid artery was exposed and ligated with a 5-0 blue nylon. After, the operated pups were placed in a 37 °C warm hypoxic chamber for 1 h to establish conditions in 8% O_2_ and 92% N_2_. Then, the operated pups were returned to their mother until they were sacrificed.

### 4.3. Human Umbilical Cord Blood Cells

hUCB was provided by the CHA Cord Blood Bank. The cryopreservation of donated hUCB was performed following the protocol of the facility. First, using fresh hUCB, plasma and the mononuclear cells were separated using the density difference for Ficoll-Hypaque (GE healthcare, Chicago, IL, USA). The mononuclear cell concentrates were washed to dextran 40 and albumin before freezing and were then cryopreserved with dimethyl sulfoxide and plasma in a controlled-rate freezer at −198 °C after collection. All human-related protocols obtained the Institutional Ethics Committee of CHA Bundang Medical Center approval.

### 4.4. Administration Condition

The number of total nucleated cells of hUCB used in the treatment was based on our previous clinical trial which was performed in patients with traumatic brain injury [49]. Before injection, the hUCB cells were washed with saline and then the number of cells was counted using a Countess (Thermo Fisher, Waltham, MA, USA). Furthermore, the dosage of rhEPO was selected based on a corresponding dose in a clinical trial for cerebral palsy that is considered safe for humans [49]. The pups were randomly divided into five groups: (1) sham group (not induced HIE); (2) HIE group (PBS, intraperitoneal injection for five consecutive days from 7 d post-HIE); (3) EPO group (rhEPO, 500 IU/kg, intraperitoneal injection for five consecutive days from 7 d post-HIE); (4) UCB group (hUCB, 3 × 10^7^/kg, intraperitoneal injection once at 7 d post-HIE); (5) UCB+EPO group (hUCB+rhEPO treatment at the same dose and schedule as the other groups).

For evaluation of the sequential short-term molecular responses 7 d after injection at P21 (14 d after HIE), the ICR mice were randomly allocated to the sham (*n* = 5), HIE (*n* = 5), EPO (*n* = 5), UCB (*n* = 5), and UCB+EPO (*n* = 5) groups. For behavioral assessment, 28 d after injection at P42 (35 d after HIE), another set of newborn mice was randomly assigned to the sham (*n* = 11), HIE (*n* = 9), EPO (*n* = 10), UCB (*n* = 10), and UCB+EPO (*n* = 10) treatment groups.

In order to confirm whether the synergistic anti-apoptotic effect of hUCB and rhEPO co-administration exerted through the Akt pathway, MK-2206 2HCl (AdooQ Bioscience, Irvine, CA, USA), an Akt inhibitor, was injected before the administration of hUCB and rhEPO. The mice were intraperitoneally injected with 100 mg/kg MK-2206 in the UCB+EPO group 1 h before hUCB and rhEPO administration for five consecutive days from 7 d post-HIE.

**Figure 8:**
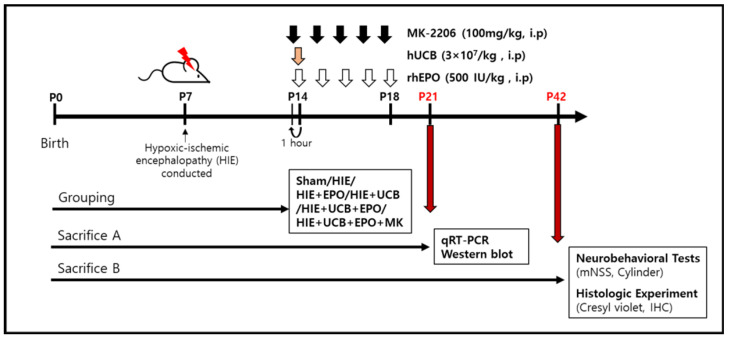
Schematic timeline for assessment of the effects of hUCB and rhEPO in ameliorating mouse brain lesions and behavioral outcomes. Postnatal day 7 (P7) mice were induced with a hypoxic–ischemic brain injury. On P14, the hUCB (3 × 10^7^/kg) was injected, administered via intraperitoneal injection, and rhEPO (500 IU/kg) was given intraperitoneally from the beginning on P14 for five consecutive days (white arrows). MK-2206 (100 mg/kg) was given intraperitoneally from the beginning on P14 for five consecutive days from 1 h before hUCB and rhEPO treatment. One week after injection, the mice were sacrificed for qRT-PCR and Western blot (red arrows). For long-term follow-up, the mice were assessed for behavioral test and histologic experiments at P42 (red arrows).

The scheme of all administrations is indicated in Figure 8.

### 4.5. Behavior Test

The neurological function of the mice was assessed using the mNSS and the cylinder test. Neurological function including motor, balance and sensory was graded on a scale of 0–14 (normal score: 0; maximal deficit score: 14) (Table 1) [27].

The cylinder test was performed to assess the functional asymmetry of the forepaw. The mice were placed in a transparent Plexiglas cylinder (diameter: 20 cm; height: 30 cm), and a total of 20 contacts was observed and the contacts each forepaw made with the cylinder wall were counted. The asymmetry score was calculated based on the formula below [29]:

$$Asymmetry\;score=\frac{impaired\;forepaw\;contact\;no.+\left(\frac{bilateral\;contact\;no.}{2}\right)}{\mathrm{total}\;\left(\mathrm{impaired}+\mathrm{nonimpaired}+\mathrm{bilateral}\right)\;contact\;no.}\times 100\left(\%\right)$$

### 4.6. Histologic Experiment

Cresyl-violet staining. The mice sacrificed after behavior tests at 4 weeks after treatment hUCB and rhEPO were dissected and perfused with 30 mL of saline followed by 4% paraformaldehyde. Coronal brain sections of 10 µm were prepared using a cryostat and stained with 0.5% cresyl-violet solution. The whole brain images of each cresyl-violet-stained brain slice were obtained under Cytation 5 (Biotek, Winooski, VT, USA). The infarct volume was calculated by a percentage for the area of the contralateral hemisphere minus the ipsilateral hemisphere using the ImageJ program.

$$Infarct\;volume=\frac{contralateral\;hemisphere-ipsilateral\;preserved\;hemisphere\;}{contralateral\;hemisphere}\times 100\left(\%\right)$$

The number of neuronal cells was manually counted for the determination of cell viability using the ImageJ program.

Immunohistochemistry. The mice treated with hUCB and rhEPO 7 days after HIE were sacrificed 4 weeks after treatment and were dissected in the same manner indicated above. The brain tissues were serially collected with a thickness of 10 µm throughout the whole brain by cryotome (Leica, Buffalo Grove, IL, USA) and mounted on glass slides. The brain tissue on the glass slides was washed in PBS and incubated for 1 h in blocking solution (2% normal goat serum, 0.5% triton X-100) at room temperature. Then, the tissues were incubated for 16~24 h with the following primary antibodies: anti-NeuN (1:1000, Novus, Littleton, CO, USA). After that, fluorescence-conjugated secondary antibodies (1:1000, Alexa 488-conjugated goat anti-rabbit) were incubated for 1 h at room temperature. Then, the tissue was mounted in ProLong Gold reagent with DAPI (Molecular Probes, Invitrogen, Waltham, MA, USA) followed by washing in PBS. To analyze the positive cell count, the ImageJ program was used after an image was obtained by fluorescence microscopy. “Color Spit Channels” was used to establish the threshold for each image. Then “Analyze Particles” was executed to analyze positive cells. Counted DAPI was referenced as relative standard and stained relative cells were counted.

### 4.7. Quantitative Real-Time Polymerase Chain Reaction (qRT-PCR)

The cellular RNA was extracted with TRIzol (Invitrogen, Waltham, MA, USA) solution from harvesting the injured right hemispheres of the mouse brains. ReverTra Ace qPCR RT Master Mix (Toyobo Co., Osaka, Japan) was used for cDNA synthesis. The quality of DNA was confirmed at 260/280 nm absorbance ratio of approximately 1.8–2.0. Quantitative real-time PCR was performed in a CFX Connect Real-Time PCR Detection System (Bio-Rad Laboratories, Hercules, CA, USA) using 100 ng RNA. β-actin is a cytoskeletal protein involved in cell structure and motility, and based cellular functions, are normalized as housekeeping genes. Primer information is shown in Table 2.

### 4.8. Western Blotting

Proteins were extracted from the affected hemisphere of the mouse brain using RIPA Lysis Buffer (Thermo Fisher, USA) with protease and phosphatase inhibitors (Sigma, St. Louis, MO, USA). The protein content of the brain tissue was quantified via the Bradford method according to the manufacturer’s instructions (Bio-Rad Laboratories, USA). Equal amounts (30 μg) of the samples were dissolved in SDS 2X sample buffer and heated at 99 °C for 6 mins. The samples were separated using SDS-PAGE and transferred to polyvinylidene difluoride (PVDF) membranes (Millipore, Burlington, MA, USA). A 5% BSA solution was used to block the membranes for 1 h at room temperature on a rocker. The housekeeping gene β-actin was employed as a loading control. The primary target antibodies were all diluted 1:1000 and incubated with anti-Nestin (Santa Cruz, Dallas, TX, USA), anti-Sox2 (Santa Cruz, USA), anti-BDNF (Santa Cruz, USA), anti-*p*-Akt (Cell Signaling, Danvers, MA, USA), anti-*p*-STAT3 (Cell Signaling, USA), anti-*p*-Erk (Cell Signaling, USA), anti-*p*-JNK (Cell Signaling, USA), anti-TLR4 (Novus, USA), anti-Bax (Santa Cruz, USA), and anti-Bcl-2 (Santa Cruz, USA) membranes at 4 °C for 16 h (overnight). A horseradish peroxidase-conjugated anti-rabbit IgG antibody (Santa Cruz, USA) at a dilution of 1:10,000 or an anti-mouse IgG antibody (KPL, Inc., Gaithersburg, MD, USA) at a dilution of 1:20,000 with blocking solution was added to the corresponding primary antibodies, followed by incubation for 1 h at room temperature. The band was detected with the ECL reagent (Millipore, USA) on the developed membranes.

### 4.9. Statistical Analysis

All datasets were analyzed for normalcy and homoscedasticity. The normality of data was controlled using the Shapiro Wilk’s test, and the homoscedasticity was checked with the Brown–Forsythe’s test. Data are indicated as the mean ± standard error of the mean. The significance of the data was determined via one-way ANOVA followed by Tukey’s multiple comparison post-hoc test. Data that did not follow a normal distribution were compared by the Kruskal–Wallis test of variance followed by Dunnett’s multiple comparison post hoc test. Analyses were performed with GraphPad Prism version 8.0.1 (GraphPad Software, San Diego, CA, USA). A value of *p* < 0.05 was considered statistically significant.

## Figures and Tables

**Table 1:** Modified neurological severity score (mNSS).

Tests	Points
Raising rat by tail (normal = 0; maximum = 3)	
Flexion of forelimb	1
Flexion of hindlimb	1
Head moved > 10 to vertical axis within 30 s	1
Walking rat on floor (normal = 0; maximum = 3)	
Normal walk	0
Inability to walk straight	1
Circling toward the paretic side	2
Falls down to paretic side	3
Beam balance test (normal = 0; maximum = 6)	
Balances with steady posture (>60 s)	0
Grasps side of the beam	1
Hugs beam and 1 limb falls down from beam	2
Hugs beam and 2 limbs falls down from beam, or spins on beam (>60 s)	3
Attempts to balance on beam but falls off (>40 s)	4
Attempts to balance on beam but falls off (>20 s)	5
Falls off, no attempt to balance or hang on the beam (<20 s)	6
Sensory tests (normal = 0; maximum = 2)	
Placing test (visual and tactile test, try 5 times and have not moved more than 3 times)	1
Proprioceptive test (deep sensation, pushing the paw against the table edge to stimulate limb muscles, try 5 times and have not moved more than 3 times)	1
Maximum Points 14	14

**Table 2:** Primers used in this study.

Gene	Forward Primer (5′-3′)	Reverse Primer (5′-3′)
TNF-α	TACTGAACTTCGGGGTGATCGGTCC	CAGCCTTGTCCCTTGAAGGAACC
IL-1 β	AAGGAGAACCAAGCAACGACAAAA	TGGGGAACTCTGCAGACTCAAACT
TGF-β	TGACGTCACTGGAGTTGTACGG	GGTTCATGTCATGGATGGTGC
IL-6	TACCACTTCACAAGTCGGAGGC	CTGCAAGTGCATCATCGTTGTTC
IL-2	GCACCCACTTCAAGCTCCA	AAATTTGAAGGTGAGCATCCTG
IL-10	ATGCTCCTAGAGCTGCGGACT	CCTGCATTAAGGAGTCGGTTAG
TrkB	CCGGCTTAAAGTTTGTGGCTTAC	GGATCAGGTCAGACAAGTCAAG
Nkx2.1	GATGGTACGGCGCCAACCCAG	ACTCATATTCATGCCGCTCGC
β-actin	AGAGGGAAATCGTGCGTGAC	CAATAGTGATGACCTGGCCGT

## Data Availability

The data presented in this study are available upon request from the corresponding author.
